# Intrathymic growing bronchogenic cyst mimicking thymoma: A case report

**DOI:** 10.3389/fonc.2023.1121321

**Published:** 2023-02-20

**Authors:** Madalina Grigoroiu, Sarah Paisley, Emmanuel Brian, Delphine Natali

**Affiliations:** ^1^ Dunarea de Jos University, Galați, Romania; ^2^ Antony Private Hospital, Antony, France; ^3^ L’Institut Mutualiste Montsouris, Paris, France

**Keywords:** bronchogenic cyst, thymoma, case report, robotic surgery, subxiphoid

## Abstract

Intrathymic bronchogenic cysts are extremely rare lesions, and the differential diagnosis with a banal thymic cyst or a solid tumor can be problematic. Thymic carcinomas arising within thymic cysts have also been reported. We report a case of radical thymectomy for a slowly growing small thymic cyst. The pathological finding revealed a bronchogenic cyst rather than a thymic neoplasm.

## Introduction

Intrathymic bronchogenic cysts are extremely rare lesions of the anterior mediastinum. The overlapping radiological characteristics with solid tumors, especially on the computed tomography (CT) scan, make the preoperative radiological diagnosis quite difficult (1, 2). Soft-tissue attenuation on chest CT scan caused by protein-rich cyst fluid can make the preoperative differentiation from solid neoplasm, particularly thymoma, very difficult (2). Enhanced CT scan combined with thoracic magnetic resonance imaging (MRI) is superior to enhanced chest CT scan or MRI alone for the preoperative differential diagnosis of thymomas and thymic cysts with diameters of <3 cm (2). The decrease in the lesion signal intensity in phase opposition at chemical shift sequences at MRI can also be useful in order to support benign lesions of the thymus (3), but the growing pattern of these doubtful lesions can lead to a surgical decision.

We present a case of a slowly growing small intrathymic cyst, which was incidentally discovered on the CT scan of the chest while evaluating a sub-centimetric pulmonary nodule. Because of its growing evolution, we strongly suspected a thymoma, and thus, a radical thymectomy was performed. The pathological finding revealed a bronchogenic cyst rather than a thymic neoplasm. In the light of this rare situation, we discuss the preoperative diagnostic issues and the therapeutic options.

## Case description

A 65-year-old male patient was referred to our hospital in July 2021, for a slow-growing intrathymic lesion, incidentally discovered in July 2015 on a routine chest CT scan for lung cancer screening. The patient was an active smoker (40 pack-years). His comorbidities were hypercholesterolemia, hypertension, and overweight (BMI 30.11 kg/m^2^). The patient has no symptoms related to myasthenia gravis and the neurological examination was normal. Routine blood test and specific blood test for thymic tumor investigation (hemoglobin, reticulocytes, serum protein electrophoresis, thyroid stimulating hormone, alpha fetoprotein, beta human chorionic gonadotropin, anti-nuclear antibodies, and anti-acetylcholine receptor antibodies) were normal except for a slight hypo-gamma-globulinemia of 7.4 g/L (N: 8–13.5) without any clinical consequences.

**Figure 1:**
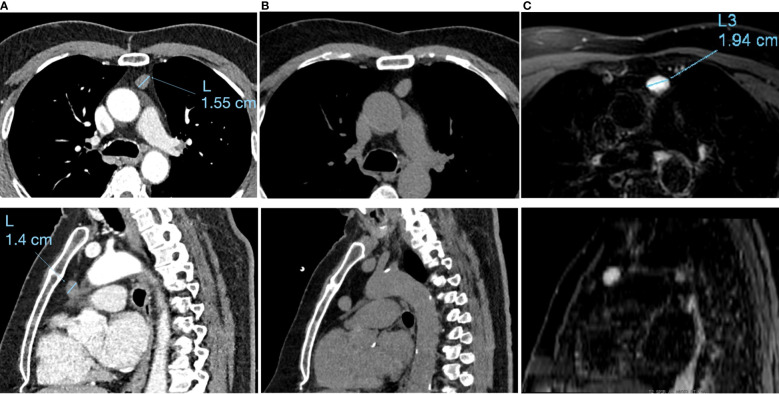
Timeline with relevant data from the episode: Axial incidences (upper range) and sagittal incidences (lower range) of the: **(A)** 2015 contrast-enhanced chest CT scan examination, **(B)** The 2021 native chest CT scan examination and **(C)** the July 2021 T2 sequence of the MRI examination.

When first discovered in 2015, the contrast-enhanced CT scan showed a 1.5 × 1.1 × 1.4 cm well-defined mass with homogeneous attenuation [Hounsfield units (HU) of 42], without mural enhancement or calcification. A slight growth was noticed on a 2018 control CT scan with a 1.7 × 1.1 × 1.7 cm (HU of 42 to 80) lesion. A further increase in size was noted on a 2021 CT scan examination. Indeed, the lesion was 1.9 × 1.2 × 1.7 cm and the patient was referred to thoracic surgery. **Figures 1A, B** show the comparative CT scan and MRI examinations.

Thoracic MRI in July 2021 (**Figure 1C**) found a homogeneous ovalar well-delimitated lesion, with T2 hypersignal and T1 hyposignal, without diffusion restriction, or signal drop on the out-phase sequence, measured at 1.9 × 1.2 × 1.8 cm. This pattern suggested a cystic lesion rather than a solid tumour. Because of its small size and the high probability of fluid content, fine needle aspiration biopsy of the lesion was considered inappropriate. However, in November 2021, due to the growing pattern of the lesion, the French national thymic tumor board (RHYTMIC) decided to propose a robotic surgical resection.

A radical thymothymectomy by a robotic extrathoracic approach (subxiphoid and subcostal approach) was performed under general anesthesia with double-lung ventilation. The patient was placed in supine position. Three-port access with CO_2_ insufflation of pleural space (pressures of 8 mmHg) was performed. After exploration of the whole chest cavity, complete thymectomy, including the lesion as well as the mediastinal fat between the two phrenic nerves, was performed.

**Figure 2:**
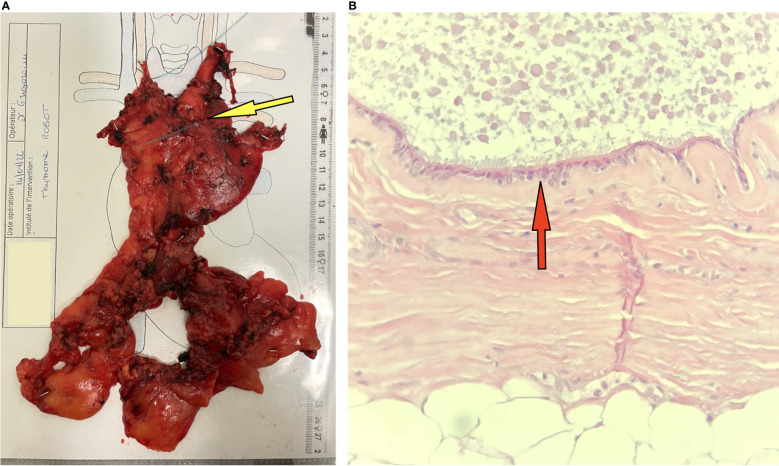
**(A)** Macroscopic aspect of the resected specimen: thymus including the thymic cyst, which is not visible. The intrathymic situation of the thymic cyst is indicated with a yellow arrow on the picture. **(B)** Microscopic aspect of the cyst: coloration HE, magnification ×400: Cyst wall lined with a unistratified layer of respiratory-type mucosa associated (red arrow) with slight fibrous and inflammatory lesions.

The specimen was retrieved through the subxiphoid incision and a 24-Fr non-aspirative chest tube was placed at the level of one of the subcostal trocar incision. The operative time was 163 min and there were no intraoperative complications. The postoperative course was uneventful. The chest tube was removed on the first postoperative day and the patient was discharged home the day after. Histologic examination showed an 18 × 14 × 8 mm cyst lesion, filled with yellow viscid fluid. The cystic wall was thin and slightly fibrous, formed by a bistratified ciliated columnar coating epithelium, with large oval nuclei, sometimes angular with flat chromatin and sometimes with a small eosinophilic nucleolus. The rest of the thymic parenchyma was in adipose involution, it did not present any anomaly (**Figure 2**). One year after the operation, the patient is doing fine, has no postoperative pain, and has resumed normal activities.

## Discussion

Intrathymic bronchogenic cysts are extremely rare, making them difficult to suspect when they are incidentally discovered on a radiologic examination. In our case, because of a growing cyst, we strongly suspected a cystic thymoma and, thus, decided to do a radical thymectomy. In the light of this case, the question arises whether we could avoid an “unnecessary thymectomy” or whether we could propose a limited resection of the thymus in case of a cystic lesions and, if so, what could be the criteria for selecting patients for a limited thymic resection.

Most of the bronchogenic cysts are located in the posterior mediastinum or intrapulmonary since they derived from abnormal germination of the embryonic foregut (4). The first bronchogenic thymic cyst was reported by Taniwaki in 1997, and there are less than 10 cases reported nowadays in the literature (5). Unusual locations of bronchogenic cysts were reported in skin, subcutaneous tissue, neck, mesentery of bowel, or the intramedullary part of the spine (1). All of them are surgically confirmed cases, but none was diagnosed as bronchogenic cyst on preoperative investigations. The different proportions of water content make differential diagnosis from solid lesions difficult on CT scan in soft-tissue bronchogenic cyst or other unusual location. Thoracic MRI examination revealed a homogeneous well-delimitated lesion, with a T2 hypersignal and T1 hyposignal, without diffusion restriction, or signal drop on the out-phase sequence, but there are no precise diagnostic criteria for bronchogenic cysts and patients are referred to the surgeon when doubt persist.

Nevertheless, surgery exposes the patient to risks and there is a concern regarding the so-called “non-therapeutic thymectomy” or “unnecessary thymectomy”, if the thymic lesion is not a cancer or the patient does not have myasthenia, since its incidence in the literature ranges from 22% to 68% (6). The postoperative complication rate in this setting ranges from approximately 10% in case of minimal invasive surgery (6) to 27% in case of open surgery (7). Moreover, the estimated inflation-adjusted costs for patients undergoing non-therapeutic thymectomy was $9,450 in a cohort of 1,796 non-therapeutic thymectomies performed from 2000 through 2009, from the Nationwide Inpatient Sample in the United States (7). Because patients in this group had a median age of 44 years, the authors suggest that it is reasonable to assume that additional cost was incurred as a result of time lost from work, even with an uncomplicated recovery. Wang et al. (8) retrospectively reviewed 1,039 consecutive patients who underwent thymectomy. Multivariable analysis indicated that patients younger than 44 years old with a precontrast/postcontrast CT value (DCT value) of less than 6 HU had a 95% probability of nontherapeutic thymectomy. Among those of nontherapeutic thymectomy, thymic cysts (11.9%, *n* = 124) were the most common lesion.

However, it is not very clear if a thymectomy for thymic cyst is an “un-necessary thymectomy”. Some surgeons advocate early surgical resection for thymic cysts in order to avoid complications, such as rupture of the cysts, malignant transformation, or compression of the surrounding organs in the mediastinum (9, 10), whereas others assert that there is no need for systematic resection due to their benign nature (11). Similar concerns are advocated for bronchogenic cysts because of possible aggravation by cyst infection, progressive growth, and spontaneous rupture. The growth of a bronchogenic cyst is thought to be the result of mucus accumulation, bleeding, infection, or malignant transformation (12). It is also known that the growth rate of bronchogenic cysts is slow in the first months of life, but that, even in the absence of complications, size increases constantly at an exponential rate (13). Malignant transformation has also been reported (12, 14). We cannot assume the reasons of the growth of the bronchogenic cyst in our case, but the clinical presentation and image findings excluded hemorrhagic and infectious causes. Histopathologic examination of the cyst showed no evidence of malignant transformation. In order to prevent complications, which are unpredictable, early surgical management of bronchogenic cysts is recommended, because after early surgery, there are fewer postoperative complications, and the hospital stay is shorter especially when surgery is performed with a thoracoscopic approach (15). As in our case, decision-making for surgery is mostly based on the increase in size of the thymic cyst (3). In this case, we considered that there was an oncological risk to perform a limited resection (a simple cyst resection), as the precise nature of a thymic cyst was impossible to determine before surgical resection. Moreover, there are no data in the literature concerning the value of the frozen section on a thymic cyst in order to decide for a limited resection in the course of the operation. Acquired thymic cysts are believed to be multilocular, contain turbid fluid or gelatinous material, and are reported to be associated with thymic tumors (16). A recent meta-analysis comparing complete to partial thymectomy in non-myasthenic patients with early-stage thymoma suggests that partial thymectomy is oncologically equivalent to complete thymectomy for non-myasthenic patients with early-stage thymoma. Moreover, there is an additional advantage of reduced postoperative complications and decreased length of hospital stay with partial thymectomy (17), but this approach is not accepted in the ITMIG guidelines (18) and need more clinical studies in order to consider it for clinical practice. There is no guidance regarding the optimal surgical approach for thymic cysts, but a thoracoscopic approach for thymectomy is also associated with a reduction in hospitalization length overall complication rate, when compared with open thymectomy (7). Moreover, robotic surgery has overcome the surgical limits of thoracoscopic surgery such as two-dimensional (2D) vision with a lack of deep perception and the use of long rigid instruments with poor maneuverability in case of fine dissection, which makes thoracoscopy difficult to adopt for surgery of the mediastinum (19). The articulating instruments specific to robotic surgery allow surgeons to operate in a limited area such as the anterior mediastinum, with fine dissection around organs and vessels when compared with thoracoscopy, and made robotic surgery a preferred approach.

In conclusion, patients diagnosed with thymic cysts fall under “unnecessary thymectomy”, especially a young patient with a unilocular cyst, containing a clear fluid within the thin wall, with a DCT value of less than 6 HU. However, in case of multilocular thymic cysts or thymic cysts presenting with symptoms, bleeding, inflammation, or growing tendency, surgical resection is indicated and partial resection should be considered. A robotic approach allows safe surgery in a limited area such as the anterior mediastinum, with reduced postoperative complications.

## Data availability statement

The raw data supporting the conclusions of this article will be made available by the authors, without undue reservation.

## Ethics statement

The studies involving human participants were reviewed and approved by CEPAR-Comite d’Evaluation des Protocoles et d’Aide à la Recherche. The patients/participants provided their written informed consent to participate in this study. Written informed consent was obtained from the individual(s) for the publication of any potentially identifiable images or data included in this article.

## Author contributions

All authors listed have made a substantial, direct, and intellectual contribution to the work, and approved it for publication.
